# Dental Students’ Perceptions of Standardized Patient Experiences Conducted Virtually: A Multi‐Year Qualitative Study

**DOI:** 10.1002/jdd.70068

**Published:** 2025-10-15

**Authors:** Jhanvi P. Desai, Michelle R. McQuistan, Lance Brendan Young

**Affiliations:** ^1^ Department of Preventive and Community Dentistry The University of Iowa College of Dentistry and Dental Clinics Iowa City Iowa USA

**Keywords:** dental communication, dental education, simulation, standardized patients, telehealth, telemedicine

## Abstract

**Purpose:**

As teledentistry becomes more common, it is important for students to develop skills in communicating with patients virtually. This study investigated students’ individual communication perceptions and preferences compared to in‐person sessions of participating in standardized patient (SP) exercises in a virtual medium.

**Methods:**

Between 2020 and 2022, first‐, second‐, and third‐year dental students completed virtual SP visits followed by a written reflection. The authors qualitatively content analyzed these assignments by first identifying discrete statements describing the impact of the virtual medium on students’ preferences, and then using a grounded‐theory constant comparative approach to identify emergent themes and subthemes. Themes were subsequently coded for valence (positive vs. negative).

**Results:**

The 485 written reflection assignments by 248 unique students yielded 189 statements for analysis. Three broad themes were identified: overall perceptions of the virtual medium, perceptions of the virtual medium's influence on students’ thoughts, and perceptions of the virtual medium's influence on students’ performance. Within these categories, nine subthemes were identified, of which three had a negative valence and six had a positive valence. The students reported mixed perceptions. A majority preferred in‐person experiences due to increased cognitive load associated with unfamiliarity with technology and multitasking while conducting the virtual exercise. A minority of students found the virtual experience enjoyable with reduced anxiety and an opportunity to broaden their communication skills.

**Conclusions:**

By anticipating and addressing student concerns proactively, and by emphasizing the value of developing teledentistry skills, instructors can help their students better understand and embrace the benefits of virtual SP training.

## 1 Introduction

Clinical communication, when done effectively, improves patient satisfaction, treatment adherence, and overall oral health outcomes [1, 2]. Conversely, poor communication increases risk of litigation and distrust between the patient and the dental provider [3]. Because teaching effective provider–patient communication can prepare dental students to become proficient oral healthcare providers, dental schools across the United States have incorporated communication training in their curricula [4]. Simulation‐based instruction is integral to dental education, and a recent study indicates more than half of United States and Canadian dental schools use standardized patient (SP) training to teach patient‐provider communication skills. Further, evidence demonstrates dental students’ communication skills improve through SP experiences [5].

According to American Dental Association (ADA), “Teledentistry refers to the use of telehealth systems and methodologies in dentistry. Telehealth refers to a broad variety of technologies and tactics to deliver virtual medical, health, and education services. Telehealth is not a specific service, but a collection of means to enhance care and education delivery” [6]. Teledentistry originated as an offshoot of telemedicine. One of its significant uses can be dated to a 1994 United States (US) military project to improve patient care as well as communication amongst dentists and laboratories [7]. With advancing technology, today teledentistry modalities can be carried out in both synchronous and asynchronous manner. These modalities can range from live video consultation (synchronous), store and forward consultation (asynchronous), remote patient monitoring (RPM), and oral health education through mobile devices (MHealth). Teledentistry was hailed as a game changer in the field of dentistry due to its contribution to patient care during the pandemic. However, findings from a 2021 CareQuest visual report indicate four reasons why teledentistry is here to stay: 1) Teledentistry ensures people have access to care in situations like infectious outbreak or natural disasters; 2) Teledentistry can help reduce costs; 3) Patients have become more receptive to teledentistry experiences and are embracing this service; and 4) It can help reduce the number of visits to hospital emergency departments [8].

Given that telehealth is becoming more common, it is important to prepare healthcare students on how to conduct telehealth visits. One way to do this is via SPs. In many non‐dental settings, SP exercises have been conducted on a web‐based platform [9, 10, 11]. An interprofessional education (IPE) study by Lempicki and Holland found that web‐based SP encounters are feasible and can help bypass logistical problems in scheduling [9]. Shortridge et al. found that nursing and allied health students had mixed perspectives about web‐based geriatric SP encounters, especially when it came to rapport building and technology [10]. In contrast, students from nursing and pharmacy programs in an eHealth interprofessional activity reported overall positive experiences regarding collaborating through telehealth technology during SP experiences [11]. A study of resident physicians found that while a majority of participants agreed that web‐based encounters with SPs were convenient and practical, 80% still preferred in‐person encounters [12].

Other non‐dental studies have also found mixed results. A study conducted among medical students found that while the online interview process was educational, some felt that the interview content was affected in the virtual medium [13]. An Observed Structured Clinical Examination (OSCE) for testing advanced communication skills in a virtual format among senior medical students showed similar qualitative themes when compared to the in‐person format. The authors noted that some students struggled with experiential learning as well as reflective observation in the virtual format [14]. Two European medical colleges found that teaching communication skills to students in their second and third year using web‐based encounters was effective, and students appreciated the one‐to‐one setting with their SPs [15]. A study conducted with pharmacy students found that the communication performance of early learners who practiced their communication skills virtually with SPs was worse compared to more senior students who practiced communication skills with in‐person SPs [16]. Pfeil and Shellhaas published a review paper in which they discussed the importance of *telesimulation*—a process whereby participants train by simulation through telecommunication [17]. One of the things highlighted in this paper is the need to know the learners involved in telesimulation exercises. The knowledge, attitudes, skills, and learner identified needs can help develop an effective, efficient simulation training.

Studies assessing the use of virtual SPs in dental school environments have also been conducted. In a dental communication study by Omar et al., online encounters were positively received by both students and SPs with the biggest challenge being technical issues [18]. Salgado and Castro‐Vale found that when faculty evaluated videoconferencing exercises between dental students and peer SPs, there were both advantages and disadvantages of online communication [19]. Patel et al. found that dental students’ comfort levels improved after virtual simulated encounters with live actor patients when compared to the pre‐experience surveys [20]. Desai et al. found dental students reported mostly negative perceptions of the impact of the virtual medium on clinician–SP interactions due to technological problems affecting patient engagement and rapport [21].

The aforementioned studies examined student performance, online processes, and faculty‐based feedback associated with web‐based SP encounters. However, few studies have assessed students’ perspectives of web‐based SP exercises in the United States, and even fewer studies have been conducted among dental students. This is the first study to qualitatively analyze students’ perceptions of virtual SP technology on individuals’ thoughts and performance. These insights regarding specific technology‐related facilitators and barriers are especially important as remote consultations become commonplace in dentistry. The purpose of the current study was to understand dental students’ perceptions and preferences pertaining to participating in SP exercises in a virtual medium. Specifically, this study sought to answer the following research questions:

RQ1: How do students perceive the overall value of standardized patient experiences that are conducted via a videoconferencing platform?
RQ2: Which specific elements of standardized patient experiences conducted via videoconferencing platforms are perceived by students as personally beneficial or problematic?

These insights regarding specific technology‐related facilitators and barriers are especially important as remote consultations become commonplace in dentistry.

## 2 Materials and Methods

This study was conducted in a dental school based in the Midwest in the United States. This study was reviewed, approved, and monitored by the University of Iowa's Institutional Review Board (IRB #202112317). As a retrospective analysis of educational materials, the study was granted by the IRB, a waiver of the elements and documentation of consent. The assignments were de‐identified before importing them into the qualitative analysis software to protect students’ identities. The SPs were acting in the capacity of paid actors portraying fictional patients, and their actual identities were not disclosed to students.

### 2.1 Course Description

All first‐year (D1), second‐year (D2), and third‐year (D3) students at the University of Iowa College of Dentistry, an educational institute in the Midwest region of the United States, are required to enroll in the three‐course series, Clinical Practice and Professionalism. These courses, which have been required curricula for more than 20 years, train students in evidence‐based practices for engaging with patients in a way which enhances oral health outcomes and patient satisfaction. Topics include rapport building, interviewing, treatment planning, motivational interviewing, health literacy, etc. D1 and D2 students participate in the first and second parts of the course series in the spring semester, while the D3 students complete the final part of the course series in the fall semester. The curriculum emphasizes patient‐provider communication in the dental clinic. Each student engages in one SP exercise during each semester of the course, for a total of three experiences across 3 years. The third experience is also a competency assessment for skills in behavioral management (United States Commission on Dental Accreditation Standard 2‐16) [22]. After each experience, a student has a 1:1 debriefing session with their SP for 10–15 min in which the SP gives the student feedback about their interaction. In addition, students complete a guided written self‐assessment about their interaction with the SP. These assessments are uploaded into the learning management system and graded on completeness. These in‐person sessions transitioned to a virtual medium (Zoom Video Communications, Inc) in Spring 2020 due to the COVID‐19 pandemic. Table 1 presents the demographic information about the students who participated in this study. The class of 2022 had one in‐person SP exercise in their D1 year (i.e., Spring 2019, prior to COVID‐19). Due to the sudden onset of the COVID‐19 pandemic in Spring 2020, the class of 2022 did not have a SP exercise in their D2 year. Instead, they evaluated video recordings of former students’ SP experiences. Neither their D1 nor D2 experiences are included in this study; however, their Fall 2020 D3 year virtual SP experience has been included. In contrast, the class of 2023 completed virtual SP experiences as D1 (Spring 2020), D2 (Spring 2021), and D3 (Fall 2021) students. All 3 years of their data are included in this study. Finally, the class of 2024 had virtual SP experiences as D1 (Spring 2021) and D2 (Spring 2022) students only. Because their D3 SP experience was conducted in person (Fall 2022), only the Spring 2021 and Spring 2022 data for the class of 2024 was included in this study. In summary, only data pertaining to virtual SP experiences were considered in this study.

**TABLE 1:** Student demographic information.

Class graduating in	Age Range (average)	Number of males	Number of females	Number of in‐person experiences	Number of virtual experiences^*^
2022	19–33 (22)	44	36	1 (D1)	1 (D3)
2023	20–33(22)	44	36	0	3 (D1,D2,D3)
2024	19–32(22)	32	50	1 (D3)	2 (D1,D2)

* Only virtual experiences were analyzed for this study.

### 2.2 Description of SPs

The SPs are actors who portray patients based on a profile that is developed for them by the faculty teaching the course. To better expand the students’ communication skillsets, SP profiles become more complex in each sequential course. SPs who participated in the virtual exercise had previously worked with dental students in the in‐person format. With the transition of the exercise, the SPs trained with the dental faculty and a technical support person to use a virtual platform to interact with students. With the onset of the pandemic, in Spring 2020 the faculty and IT specialist met with the SPs individually for an hour to help with technical and transition related training. The training described the case vignettes and provided suggestions regarding how to interact with the students in a virtual setting (e.g., use verbal emotional reactions in addition to relying on non‐verbal cues that may be hard to see virtually; to simulate eye‐contact, make “eye contact” with the computer camera rather than the student's face on the screen). In addition, the training also focused on calibration using a grading rubric and provided suggestions on how to troubleshoot potential technical issues. In the following years, as using Zoom became more commonplace, the faculty met with the SPs at the start of the exercises for a revision training and calibration. After the training sessions, the faculty checked in with the SPs weekly via email to address any issues that may have developed. The SPs were given dedicated time at the end of the student's experience to provide feedback to the student.

### 2.3 Conducting SP Exercises on a Virtual Platform

Like all other courses in the dental school, the lectures of the Clinical Practice and Professionalism course series transitioned to the virtual medium during the COVID‐19 pandemic to prepare students for their SP exercises. The lecture content of this was similar to the in‐person sessions. In addition to this students were given detailed instructions about using features on the Zoom platform, such as sharing screens and ability to record, to help prepare for the video conferencing exercise. The dental faculty created a schedule such that each student in each course spent 1 hour with a SP. To accommodate an entire class, the SP experiences occurred over several weeks in the semester. The first 45 min of each simulation were dedicated to the student–SP interaction, and the remaining 15 min were allocated for debriefing and feedback from the SP. The faculty matched each student to a SP from a pool of six to eight actors. Care was taken to ensure that no SP profiles were repeated with the same student over the 3‐year curriculum. Zoom allows for “Breakout rooms” where participants can be separated from the main session to allow for distraction‐free engagement. The faculty created six to eight breakout rooms during each scheduled SP experience so that private 1:1 student–SP sessions could be conducted simultaneously while allowing each student to independently record their SP experience. Through this mechanism, students could experience a simulation mimicking a synchronous teledentistry encounter.

### 2.4 Data Collection

Participants in this study included all of the students enrolled in the Clinical Practice and Professionalism course series from May 2020 through April 2022. A total of six sections were included in this study: D1 in Spring 2020 and 2021; D2 in Spring 2021 and 2022; and D3 in Fall 2020 and 2021. This consisted of 248 students. The D2 Spring 2020 course did not include any SP exercises due to the rapid COVID‐related shutdowns, while the D1 Spring 2022 students completed the SP exercise in‐ person. The SP–student encounters that were included in this study were the ones conducted via Zoom. The students and the live actor SPs conversed in a dynamic manner that was recorded so that students could self‐assess their performance after the SP interaction. The graduating classes of 2022, 2023, and 2024 comprised the study participants.

The 485 written self‐reflection assignments were assessed by the three faculty who delivered the courses and co‐authored this article. Data were extracted from students’ responses to prompts on their self‐assessment assignments. These prompts included a self‐evaluation of their interaction with the SP. They also included questions which ascertained their beliefs and recommendations about the online exercise. For example, one question asked, “How did the teledentistry context make the encounter easier or more difficult than face‐to‐face?” Another prompt asked, “What recommendations would you make to improve the [virtual] SP experience in the future?”

### 2.5 Data Analysis

The authors conducted a qualitative content analysis of the data. Students’ self‐assessment submissions were imported into QSR International's NVivo (Release 1.6.1) software. Responses to the prompts about students’ perceptions, recommendations, and preferences regarding the use of a virtual medium to complete the SP exercises were reviewed. Initial coding focused on manifest content including the gender (female: F, male: M) and academic year of the student (i.e., D1, D2, or D3) as well as the semester (Fall or Spring) and year (2020, 2021, or 2022) in which they participated in the course.

The authors had previously read and graded the assignments from their classes when the assignments were initially submitted, so they were already familiar with the types of feedback students provided. They determined at the outset that the students’ responses about the virtual medium should be analyzed for themes as well as the valence of the comment (i.e., positive, or negative). After coding manifest content, the authors divided the dataset, and each author open coded latent content in approximately 50 of the submissions from their students. Each worked independently, but working from a common dataset in a shared program meant they were able to view and apply initial codes generated by the others. This process entailed node memos defining the code and providing exemplars.

The thematic analysis was done in accordance with the grounded theory, constant comparative approach per Corbin and Strauss [23]. The constant comparative method was used during this process to iteratively determine whether an identified theme (a) fit an existing code, (b) required a modification of the code's definition, or (c) required creation of a different code. The unit of analysis was a theme, or an expression of an idea [24]. Themes were expressed in a statement—a clause, sentence, or paragraph, depending on the student. Most statements communicated a single theme, but in cases in which students communicated multiple ideas in a single statement, multiple themes were assigned in order to accurately represent meaning. During open coding, each theme was assigned one, and only one, valence, either positive or negative, as students did not express ambivalence about the ideas coded as themes.

After initial coding of the data, the authors met in person to discuss the themes that emerged, to reconcile different understandings, and to create categories distinguishing themes from subthemes. Using the resulting shared codebook, they then independently coded the remainder of the dataset, guided by project memos available to all, by adjustments to the node definitions, and by face‐to‐face meetings at regular intervals over 3 months. These meetings focused on integration and validation of the coding frame and ultimately led to the development of the tables presented in this article.

The themes were further categorized as dyadic factors, where student–patient interactions were affected by the virtual medium, and individual factors, where students’ attitudes and preferences about the virtual exercise were noted. Dyadic factors were described in a separate study [20]. The current study presents information related to the individual‐level factors.

## 3 Results

The demographic details of the students of the three classes that participated in the virtual SP exercises are described in Table 1. Male students comprised 55% of the classes of 2022 and 2023, while female students comprised of 61% of the class of 2024. A total of 485 written reflection assignments were completed by 248 students. The number of reflections is larger than the number of students because some students completed virtual SP exercises in sequential semesters. The written reflections yielded 189 statements addressing the virtual medium's impact on individual performance or preferences. When coding was completed, three emergent themes had been identified and further divided into nine subthemes. Three of these subthemes had a negative valence, and the remaining six subthemes had a positive valence. The three themes were: overall perceptions of the virtual medium, perceptions of the virtual medium's influence on students’ thoughts, and perceptions of the virtual medium's influence on students’ performance.

Table 2 presents students’ overall perceptions of the virtual medium. It includes general perceptions of the virtual medium—liked versus disliked—without detailing specific ways the virtual experience affected their thoughts or behaviors. Fifty of these global assessments had a negative valence, and 14 had a positive valence. One reason that students reported having a negative experience was related to a lack of familiarity with the technology. Some students felt that the interaction over the virtual medium was “not real,” while other students preferred in‐person interactions to web‐based interactions. For example, a D1 female student in the Spring 2021 semester reported that, “The Zoom format limited the experience, I would prefer to interview the patient personally, it is going to be more realistic experience beside the advantage of skipping the technical issues and the setup differences that students have.” A minority of students, however, reported a positive general outlook towards this exercise. One student wrote, “I think the online format worked pretty well,” (Spring 2021/D2/M).

**TABLE 2:** Students’ overall perceptions of the virtual medium's influence on the standardized patient exercise.

Subthemes	Description	Examples
− Negative experience (*n* = 50)	Students disapproved for various reasons such as unfamiliarity with technology, unrealistic encounter, and personal preference.	*I thought the online aspect of the encounter made things infinitely more challenging*. Spring 2022/D1/M *I just wish this exercise could have been in person as it would have made it seem more “real.”* Spring 2021/D1/F *I think the online context made the encounter much more difficult because I am not very familiar with zoom meetings*. Spring 2022/D2/F
+ Positive experience (*n* = 14)	Students approved of the virtual medium overall, with advantages not specified.	*I feel this exercise is a wonderful entry level student clinician patient interaction*. Spring 2020/D1/F *I thought the teledentistry appointment went well and was smoother than I originally expected*. Fall 2021/D3/F

Table 3 reports students’ perceptions about how participating in a synchronous online interaction with a SP affected their thinking and frame of mind. One of the negatively valenced subthemes, *cognitive overload*, indicated multi‐tasking over the computer led to student distraction. For example, “I did not use any presentation aids besides x‐rays because I was constantly worried about extra things interfering in my zoom session,” (Spring 2021/D2/M). *Increased anxiety*, another negatively valenced subtheme, attributed student nervousness to the virtual medium. A D2 male student in Spring 2022 pointed out: “I feel the online context made it more difficult for me to feel comfortable with the patient, especially in the beginning. While reviewing the video, I did become more comfortable over time, but the beginning I struggled to not be nervous and awkward. In person, I feel more comfortable connecting with the patient.” In contrast, *reduced anxiety* was a subtheme endorsed by a few students who found the virtual medium less threatening. One student wrote, “I really enjoyed the zoom aspect and thought it was a little less nerve racking then in person,” (Spring 2020/D1/M). For this positively valenced subtheme, it is worth noting that all who said they were less nervous because of the virtual medium were D1 students who had no previous experiences communicating with SPs.

**TABLE 3:** Students’ perceptions of the virtual medium's influence on their thoughts.

Subtheme	Description	Examples
‐ Cognitive overload (*n* = 30)	Students found multitasking challenging in the virtual medium.	*The teledentistry context made it difficult to access my notes, the chart, and radiographs all while also maintaining eye contact with my patient. As someone with dyslexia, I had a very difficult time managing all of the different screens with different sets of information*. Fall 2021/D3/F
‐ Increased anxiety (*n* = 27)	Students were more nervous or apprehensive in the virtual medium.	*My only recommendation is to find a way for us to do future patient experiences in a clinical setting. It was very stressful to add technology difficulties when many of us were already nervous about it being our first patient experience. It was also hard to tell which struggles of mine were just zoom related and easy fixes in the clinic*. Spring 2021/D1/F *I think that the online context might contribute to some of the anxiety these assignments bring me when compared to face‐to‐face interactions. I do not think that this assignment adequately reflected my true skill in this aspect as my anxiety kind of took control, but I do believe that this skill could still use some work on my part*. Spring 2022/D2/F
+ Reduced anxiety (*n* = 25)	Students were less nervous or apprehensive in virtual medium and gained confidence.	*I think having this virtual format was actually very beneficial because I think I would be more tense if my first SP was in‐person*. Spring 2020/D1/M *Personally, I enjoyed the virtual format more than I think I would have in person. I was less nervous and it helped to gain some confidence for the next time we do this in person*. Spring 2020/D1/M

Table 4 describes students’ perceptions on how the virtual medium influenced their performance. This theme has four subthemes, all positively valenced, describing skills that students could build and resources that they could conserve with the transition of the exercise to an online format. The first subtheme, *increased experience*, indicated that exposure to the exercises over the virtual medium helped students develop the capability to manage patients for a virtual visit. The second subtheme, *enhanced ability to self‐assess*, indicated students were able to evaluate their own skills by reviewing the recordings, which were easy to produce in the virtual medium. For example, “I just didn't know what to expect. But the fact it went well, and I could re‐watch myself be in the clinician role was very helpful for me personally. I can be honest with myself with the good and the bad, to be a better communicator with my future patients,” (Spring 2021/D1/F). The third subtheme, *ability to practice telehealth visits*, highlights that the dental students appreciated having some exposure to conducting an online interaction with patients since virtual visits are becoming more common in healthcare. A student wrote, “I think the exercise was informative and really good since teledentistry/telehealth is more than likely going to become a new norm moving forward,” (Fall 2020/D1/M). The final valence, *enhanced preparation*, focused on the effective use of time and conserving clinic resources. A D2 student wrote, “I think the only advantage teledentistry serves is to save time for both of us (me setting up the clinic, her getting here) and money (preparing the room and PPE for me, travel costs for her),” (Spring 2021/D2/M). No negatively valenced subthemes emerged in the theme reflecting the virtual medium's impact on performance, suggesting the perceived negative impacts on thought processes (second theme) were not perceived to have translated into compromised performance (third theme).

**TABLE 4:** Students’ perceptions of the virtual medium's influence on their performance.

Subtheme	Description	Examples
+ Increased experience (*n* = 11)	Students learned how to manage a virtual patient visit and were more comfortable with the exercise.	*After completing multiple standardized patient exercises via teledentistry format in the past years, I feel I can confidently say that I am getting more comfortable communicating with patients via the “on‐line” format and look forward to always gaining more experience in these situations!* Fall 2021/D3/F *I thought this teledentistry visit felt more natural than a lot of the others I have had throughout school. This may be due to increased patient exposure. I used radiographs and a treatment plan with costs that I had typed up. I thought that they helped a lot, especially the typed treatment plan*. Spring 2021/D2/F
+ Enhanced ability to self‐assess (*n* = 8)	Simple and close‐up recording captured important details of the student's performance.	*I did like the zoom format because not only was it recorded which allowed me to hear myself, but it also allowed me to see my facial expressions and where my eyes were*. Spring 2020/D1/M *I think it was very helpful to have audio and video of myself. In the past I never really reviewed these interactions, but I found it helpful to be able to look back and also listen*. Fall 2020/D3/F
+ Ability to practice telehealth visits (*n* = 9)	Students could practice honing skills to communicate via a virtual platform	*Although there are many benefits and pitfalls to the experience and its potential implementation with teledentistry. I feel it is good practice for me because I [aim] to implement teledentistry service into my own office*. Spring 2021/D2/M *The virtual experience was a good learning experience and I am sure e‐visits will be more common in the future, so I am glad I was able to practice with a standardized patient*. Spring 2020/D3/F
+ Enhanced preparation (*n* = 15)	The student was able to use time well and did not have to set up operatory, use protective equipment, etc.	*I believe the teledentistry appointment was made easier since we did not have to clean or set up our units with barriers, instrument cassettes, and other supplies. This saved valuable time, all while providing the patient with a rather similar experience as they would get in the dental office*. 2021/D3/M

## 4 Discussion

This study analyzed students’ reflections on SP experiences conducted via Zoom to identify aspects of these virtual experiences students preferred or did not prefer. The first research question about students’ perceptions of the value of virtual SP experiences was answered by the 64 thematic statements regarding the *overall* experience: the negative statements outnumbered the positive statements 3‐to‐1 as indicated in Table 2. This finding suggests a general dislike of the virtual medium for conducting SP experiences, but this conclusion is complicated by the more specific subthemes, which answer the second research question on the specific elements of the virtual encounters students found beneficial or problematic. When examining the subthemes across all three tables, only three of these subthemes were negative, while six were positive. Although in total 107 statements were negative compared to 82 that were positive, these numbers are heavily influenced by the number of statements in the overall experience. Excluding the statements from Table 2 (overall experience) demonstrates that 57 statements from Tables 3 and 4 were negative compared to 68 that were positive. Several explanations and implications are worth considering in interpreting these findings.

First, while most general statements regarding the virtual platform experience were negative, a notable minority were positive. Further, when specific aspects of the experience were mentioned, most statements were positive. These mixed results align with other studies which have reported some students prefer face‐to‐face while others are more approving of virtual encounters [9, 13]. During the early onset of the COVID‐19 pandemic, many students were unfamiliar with virtual platform software, thus it is understandable that adding this technology may have caused negative emotions. The pandemic also brought many stressors due to the sudden shift to remote learning, which disrupted established routines and social connections and contributed to feelings of isolation and stress [25]. Although online platforms offered flexibility and accessibility, many students reported decreased engagement and reduced interaction with instructors and peers [26]. These challenges along with the emotional and logistical strain of the pandemic could also have influenced the overall negative perceptions among students about this virtual exercise.

As the lockdown continued, “virtual platform fatigue” lead to people contrasting the deficiencies of the virtual medium with the virtues of live, in‐person engagement. In the case of dental students, they were not receiving the in‐person patient encounters they had expected in dental school, perhaps leading to further disappointment focused on the virtual medium. If the virtual SP experience had been an adjunct, alternative, or follow‐up to an in‐person visit, rather than an imposed substitute, perhaps general attitudes would not have been as negative. As dental schools incorporate teledentistry into their curricula, they should frame the experiences in a way that acknowledges the preference for face‐to‐face encounters but emphasizes virtual encounters’ utility in certain contexts like rural or inner‐city areas that lack dental providers or specialists. Instructors could also emphasize that some providers (and patients) prefer virtual encounters. These perceptions also emphasize the importance of introducing teledentistry communication skills early in the curriculum with dental communication skills so as to not view them as “other” or “less than” or “more challenging” option of patient communication.

Second, numerous students did specify aspects of the virtual SP experience that they disliked, so the general disapproval should not be dismissed. Our previous analysis of this dataset found 9 negative subthemes of 13 describing the impact of the virtual medium on patient interaction [20]. In the current study of individual students’ thoughts and performance, only two specific negative subthemes were described: increased cognitive load and increased personal anxiety. These are distinct subthemes which are likely mutually‐reinforcing. According to Bong et al., appropriate levels of cognitive load help while excess levels can impair student performance in simulation exercises [27]. According to the experiential learning theory, student learning through reflections and cognitive work during role‐play and active exercise is effective. Every student perceives the cognitive load differently; therefore, educators may need to innovate their teaching techniques to allow for student engagement, especially in a virtual setting [14].

We found nearly as many reports of reduced anxiety (25) as increased anxiety (27), which may reflect student variability in the ability to manage cognitive load. Comments referenced multi‐tasking and technology‐related stress, both of which could be mitigated in the future by consolidating patient information, history, radiographs, etc., and employing a more user‐friendly software or providing technology specific training to prepare students for teledentistry encounters. This is similar to the findings in the study by Choi et al. where the authors noted additional cognitive load from the virtual setting as well as difficulty in engagement in an online format due to technological difficulties and distractions, which created some challenges in student learning [14]. Pfeil and Shellhaas found that having participant learners observe short videos gave them an opportunity to model best practices in preparation for a simulated encounter. This in turn can help reduce student anxiety and stress during the actual virtual encounter. In addition, briefing the learner about the patient they will be seeing, with clear goals about the encounter and time duration, can reduce student frustration and anxiety [17].

Third, when considering specific aspects of the virtual medium, the positive assessments outnumbered the negative in both number of subthemes and number of comments. In contrast to the reports of increasing anxiety, some D1 students who had previously never participated in any simulation‐based communication exercises reported that the virtual experience reduced their anxiety. Notably, this subtheme was absent in D2 and D3 statements. Perhaps by their second year, students were less nervous because they had already completed one visit simulation and had been providing dental care to patients in‐person for several months in the D2 and D3 year. In other words, greater familiarity with patient visits and with virtual medium technology may have lowered the baseline anxiety level so that a(nother) virtual patient visit would not lower it further. In addition, evidence of the “mere exposure effect” suggests repetition and familiarity increases liking [28, 29]. This could be because repetition may reduce extraneous load, lowering anxiety, and improving cognitive efficiency accounting for students to like remote SP experiences [13, 30]. Unless students have the opportunity to repeatedly engage with SPs in a virtual medium, they may never reach the point of liking it. This is problematic given that it has been suggested that students do not receive adequate training prior to providing telehealth services [31]. If telehealth visits are going to become more common in dentistry, it will be important to provide intentional virtual SP experiences that address the various negative themes presented in this study. In addition, faculty can prepare the students for the negative factors and brainstorm ways to minimize them. Faculty can also emphasize the positive factors as benefits of doing the SP exercise virtually which can expand the student's skillset. It is important to highlight to students that teledentistry skills can benefit them by preparing them to continue patient care during disasters as well as give them an opportunity to expand their practice's services and/or patient pool.

This study did find positively valenced subthemes related to the utility of the experience for training and personal skill development. These findings are consistent with previous studies that reported positive evaluations of the intimate context afforded by virtual visits [15] and the utility of video recording and reviewing [32, 33], which is somewhat easier to accomplish in virtual visits compared with in‐person visits. Because virtual visits have lower transportation and scheduling barriers than in‐person SP exercises, the SP pool may expand to include individuals with more diverse identities than the in‐person pool allows, such as including more participants with physical disabilities, etc. Although virtual SP experiences should not supplant face‐to‐face experiences, there is value in expanding students’ skillsets by including virtual SP visits after they have mastered the in‐person experience. Further, faculty can help train SPs for teledentistry visits to make the experience as realistic as possible to what students may do in their practices in the future.

To our knowledge, this is the first study to analyze dental students’ perceptions of SP experiences in a virtual medium across multiple years and across multiple stages of training. However, there are some limitations of the study. The authors who analyzed the student data also served as instructors for some of these students. Bias is a possibility; however, several measures were implemented to mitigate this risk. All coders were granted access to the complete dataset in NVivo, allowing them to run queries on any given code and view its application across the entire dataset, even though each coder was responsible for only a subset of the data. In addition, the team collaborated using a shared codebook within NVivo, which included definitions and exemplars to promote consistent coding practices. Regular in‐person meetings were held, where they made observations about their process, noted patterns in the text, and reviewed one another's work to see where emergent themes were identified and (later) how those themes were applied/counted as codes. Because the written reflections were also graded assignments, students may have reported more positive impressions of the experience than they felt, although the large majority of negative perceptions is evidence to the contrary. In addition, no comparative analysis was done among students in the same or different years which can be considered as a limitation of the study. Although some students speculated about patients’ preferences (e.g., the cost saving of not traveling), we did not formally elicit patients’ insights. They may have offered context and a broader perspective than that of students. As with all qualitative research, the findings are specific to the individuals, times, and places studied. The themes that emerged as perceived by students in one predoctoral dental program may not be representative of other students in other contexts.

Future research should expand understanding of the benefits and shortcomings of SP experiences in a virtual medium by studying different students in different programs within the United States and globally. Additional methods should also be used, including survey and experimental methods which can produce quantifiable data subject to significance testing. Because no SP experiences can occur without active participation of SPs, their perceptions about these experiences should also be studied.

We recommend instructors provide students with strategies for managing anxiety and cognitive overload both when teaching students about teledentistry and when preparing them for a virtual SP encounter. A minority of students approved of the experience and specifically mentioned reduced anxiety and opportunities to broaden and deepen their patient communication skillsets. Instructors should also develop learning objectives associated with teledentistry lessons and experiences that amplify the value to students of expanding their skillsets and expanding their future patient pool by learning to engage with patients online.

## 5 Conclusion

As teledentistry becomes more common within dentistry, dental schools may begin to incorporate virtual SP experiences within their curricula. This study revealed that most students generally preferred in‐person experiences compared to virtual experiences due to increased anxiety and cognitive overload being associated with the virtual experience. By anticipating and addressing these concerns proactively, and by emphasizing the value of developing teledentistry skills, instructors can help their students better understand and embrace the benefits of virtual SP training.

## Author Contributions

All of the three authors have made significant contributions to the study conception, design, data collection, analysis, result interpretation, and manuscript preparation. All authors have reviewed the results and approved the final version of the manuscript.

## Ethics Statement

This study was reviewed and approved by the University of Iowa's Institutional Review Board (IRB #202112317).

## Conflicts of Interest

The authors declare no conflicts of interest.

## Data Availability

The data that support the findings of this study are available from the corresponding author, Dr. Jhanvi Desai, upon reasonable request.
